# Imaging and biophysical modelling of thrombogenic mechanisms in atrial fibrillation and stroke

**DOI:** 10.3389/fcvm.2022.1074562

**Published:** 2023-01-16

**Authors:** Ahmed Qureshi, Gregory Y. H. Lip, David A. Nordsletten, Steven E. Williams, Oleg Aslanidi, Adelaide de Vecchi

**Affiliations:** ^1^School of Biomedical Engineering and Imaging Sciences, King’s College London, St. Thomas’ Hospital, London, United Kingdom; ^2^Liverpool Centre for Cardiovascular Science, University of Liverpool and Liverpool Heart & Chest Hospital, Liverpool, United Kingdom; ^3^Biomedical Engineering, University of Michigan, Ann Arbor, MI, United States; ^4^Centre for Cardiovascular Science, The University of Edinburgh, Edinburgh, United Kingdom

**Keywords:** atrial fibrillation, stroke, computational cardiology, left atrial appendage, medical imaging, Virchow’s triad, thrombus formation

## Abstract

Atrial fibrillation (AF) underlies almost one third of all ischaemic strokes, with the left atrial appendage (LAA) identified as the primary thromboembolic source. Current stroke risk stratification approaches, such as the CHA_2_DS_2_-VASc score, rely mostly on clinical comorbidities, rather than thrombogenic mechanisms such as blood stasis, hypercoagulability and endothelial dysfunction—known as Virchow’s triad. While detection of AF-related thrombi is possible using established cardiac imaging techniques, such as transoesophageal echocardiography, there is a growing need to reliably assess AF-patient thrombogenicity prior to thrombus formation. Over the past decade, cardiac imaging and image-based biophysical modelling have emerged as powerful tools for reproducing the mechanisms of thrombogenesis. Clinical imaging modalities such as cardiac computed tomography, magnetic resonance and echocardiographic techniques can measure blood flow velocities and identify LA fibrosis (an indicator of endothelial dysfunction), but imaging remains limited in its ability to assess blood coagulation dynamics. In-silico cardiac modelling tools—such as computational fluid dynamics for blood flow, reaction-diffusion-convection equations to mimic the coagulation cascade, and surrogate flow metrics associated with endothelial damage—have grown in prevalence and advanced mechanistic understanding of thrombogenesis. However, neither technique alone can fully elucidate thrombogenicity in AF. In future, combining cardiac imaging with in-silico modelling and integrating machine learning approaches for rapid results directly from imaging data will require development under a rigorous framework of verification and clinical validation, but may pave the way towards enhanced personalised stroke risk stratification in the growing population of AF patients. This Review will focus on the significant progress in these fields.

## 1. Introduction

Atrial fibrillation (AF) affects almost 50 million people worldwide and accounts for up to one third of all ischaemic strokes. Its diagnosis and management pose a substantial burden on healthcare systems, warranting novel clinical approaches, including those for stratifying patient stroke risks (1, 2).

The most common and validated risk factors for stroke and bleeding have been used to formulate simple clinical risk scores based on patient characteristics and comorbidities, such as the CHA_2_DS_2_-VASc and HAS-BLED scores. Such empirical approaches are clinically effective for high-risk AF patients but remain suboptimal for other cohorts (3, 4). A key limitation of these scores is that they focus mainly on the role of pre-existing conditions as determinants for future stroke risk, without considering anatomical and functional metrics from imaging and clinical exams. However, the effectiveness of treatment is contingent on a reliable and personalised approach to risk stratification that is based on the assessment of all mechanisms contributing to the prothrombotic state in AF.

**FIGURE 1:**
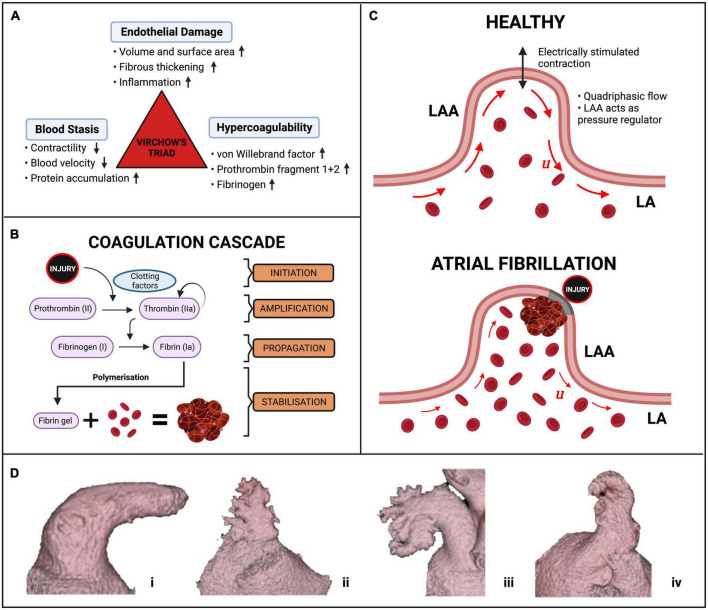
AF and thrombus formation. **(A)** The components of Virchow’s triad for thrombus formation. **(B)** The simplified coagulation cascade. **(C)** Schematic of thrombus formation in a diagram of an LAA. The red arrows represent the magnitude and direction of blood flow velocity u inside the LAA in normal conditions (top) and during AF with accumulation of erythrocytes (bottom). **(D)** The four morphologies of the LAA, **(i)** chicken wing, **(ii)** cactus, **(iii)** broccoli, **(iv)** windsock (16).

Current risk scores may be partially improved by multi-modal cardiac imaging, which can capture left atrial (LA) shape and motion, cardiomyopathies and, via advanced imaging techniques such as 4D Flow MRI, intra-cavity blood flow characteristics. Concurrently, biophysical computational modelling has emerged as a powerful tool for personalised simulation of the three main phenomena known to influence thrombogenesis (5–9)—Virchow’s triad of blood stasis, hypercoagulability, and endothelial dysfunction (Figure 1A).

This review will present the mechanisms of thrombus formation, the role of cardiac imaging in detecting LA thrombi, and the state-of-the-art computational models that have been developed for each aspect of Virchow’s triad. This will be followed by an overview of the outstanding challenges and proposed directions of this growing field to show how integration of novel modelling techniques with routinely available imaging protocols can improve current knowledge and stroke risk stratification in AF patients.

### 1.1. Mechanisms of thrombus formation in AF

Thrombus formation is mediated by the four-stages of the blood coagulation cascade, centred around the generation of the key enzyme thrombin (Figure 1B). In the initiation phase, injury to the endothelial lining exposes tissue factor (TF) and releases plasma von Willebrand Factor (vWf), forming trace amounts of thrombin from its precursor, prothrombin. The subsequent amplification and propagation phases lead to platelet activation near the injury, initiating large-scale thrombin generation which cleaves plasma fibrinogen to form insoluble fibrin monomers. In the ensuing stabilisation phase, polymerisation of fibrin monomers creates a crosslinked fibrin net that traps platelets and red blood cells (erythrocytes). This solidification of blood to a fibrin and erythrocyte-rich clot (rouleaux) finally generates a haemostatic plug over the area of endothelial injury to facilitate healing (10). AF alters this fine-tuned coagulation system and induces a pro-thrombotic state by the mechanisms outlined in Virchow’s triad (see details below), significantly increasing the risk of stroke (11).

### 1.2. The left atrial appendage

The primary site of interest is the LA appendage (LAA), a muscular extension to the LA, in which over 91% of AF-related thrombi are formed (Figure 1C) (12–15). AF-related thrombi originating from the LAA are larger and have higher risk of mortality than other thromboembolic sources but can be prevented by oral anticoagulation (OAC) (2). The LAA has four clinically defined morphologies—chicken wing, windsock, cactus, and broccoli (Figure 1D)—each with thin trabeculated endothelial walls and varying risks of thrombogenesis associated with their size, shape and blood flow velocities (16–18). The LAA, especially the “broccoli” morphology, is most susceptible to pathological thrombus formation as it demonstrates all three aspects of Virchow’s triad.

### 1.3. Virchow’s triad

Atrial fibrillation induces blood stasis by facilitating uncoordinated electrical activations which impair LA contractility and lead to reduced blood flow velocities, particularly in the LAA (19, 20). During AF episodes, increased blood residence times and peak flow velocities of <20 cm/s inside the LAA facilitate accumulation and interactions between procoagulant factors, platelets and erythrocytes in this region (Figure 1C) (21–24).

Hypercoagulability in AF patients is expressed by abnormal levels of vWf, thrombin-antithrombin complex (TAT), plasma fibrinogen and fibrin D-dimer, all of which are associated with increased propensity for thrombus formation in the LAA (11). Studies suggest the hypercoagulable state is induced by the onset of acute AF, with as little as 15 min of AF being sufficient to increase thrombin generation (25–27). Markers of endothelial dysfunction, such as inflammation and matrix remodelling, may be linked with this altered coagulability, but the precise mechanisms remain unclear (28).

Finally, endothelial dysfunction is caused by abnormalities in the endothelial lining which can trigger thrombus formation by release of thrombogenic proteins in the LAA (29). The reduced cardiac output due to the irregular contractility of the myocardium during AF is often compensated by LA volume dilation and stretching, resulting in the deposition of interstitial fibrosis, and are recognised predictors of all-cause mortality and ischemic stroke risk (30). Studies also suggest that fibrosis may promote the formation of miniature thrombi on the rough endocardial surface and create more arrhythmogenic substrate thereby perpetuating AF and its associated risk of stroke (31, 32).

## 2. Cardiac imaging for AF and stroke risk

Cardiac imaging plays a crucial role in assessing stroke risk through the detection of anatomical and functional anomalies associated with LA thrombi and assessment of thrombogenic cardiomyopathies induced by AF (33). Although clinically applicable imaging modalities for the assessment of hypercoagulability have not yet been developed, established imaging techniques are routinely used to identify both blood stasis and markers of endothelial dysfunction. However, the thin walls of the LA, small diameter of pulmonary veins (PVs) and intricate, multi-lobar/oriented shapes of the LAA make imaging of these anatomical structures challenging.

### 2.1. Blood stasis

**FIGURE 2:**
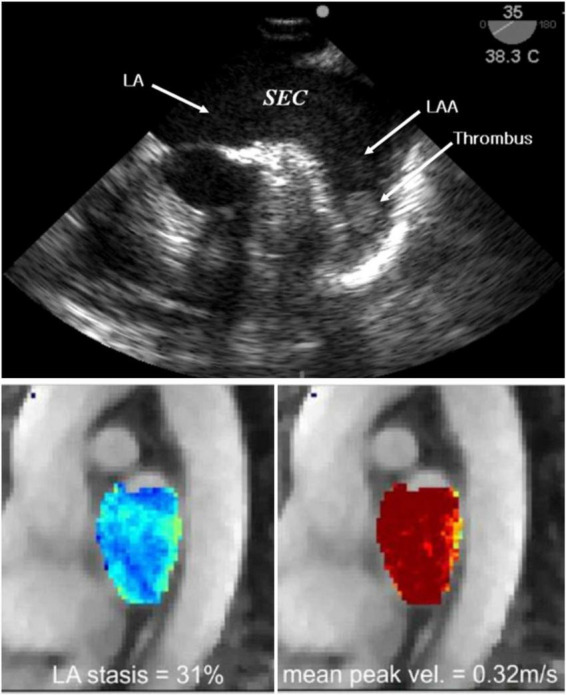
Imaging of blood stasis. 2D TEE imaging of the LA and LAA. Spontaneous echo contrast (SEC) can be seen with a dense smoke near inside the LAA indicating presence of a thrombus **(top)** (123). 4D Flow MRI results indicating blood stasis within the LA cavity and peak velocities at the centre and walls of the LA **(bottom)** (43).

For decades, 2D transoesophageal echocardiography (TEE) has been the gold standard modality for detecting the presence of LA thrombi and evaluating the appearance of spontaneous echo contrast (SEC), a predictor of flow stasis strongly correlated with LA thrombus formation, as shown in Figure 2 (34). SEC is generated by ultrasonic backscatter from erythrocytes aggregation, usually mediated by fibrinogen, and is present in ∼50% of AF patients and 90% of patients with thrombi. The severity of SEC is graded based on its visual appearance to assess the subsequent risk of LA thrombus with TEE having an excellent specificity of 100% and a sensitivity of 99% (35). However, the semi-invasive oesophageal intubation is time-consuming and contraindicated for some patients (33). Non-invasive, speckle-tracking Doppler echocardiography has been employed to evaluate LA mechanical function via myocardial strain (36, 37). This metric measures the contractility/deformation of the LA wall, which is severely impaired during AF and directly influences intracardiac blood motion, potentially leading to stasis (38).

Cardiac magnetic resonance (CMR) imaging and cardiac computed tomography (CCT) are two non-invasive alternatives to TEE for the identification of thrombi (39). A meta-analysis and systematic review of clinical trials assessing LA thrombi by CCT showed a mean sensitivity and specificity of 96 and 92%, with dual-enhanced CCT protocols able to increase the latter to 100% (27, 28). A similar meta-analysis showed that contrast-enhanced CMR angiography has a specificity of 95.2% and sensitivity of 66.7%, rising to 99.2 and 100%, respectively for delayed-enhancement CMR with long inversion time (40, 41).

Cardiac magnetic resonance and CCT allow visualisation of the LA anatomy including the PVs, which can provide useful anatomic information to guide AF ablation and LAA occlusion therapy (33). CCT has a spatial resolution of 0.5 mm in the x and y (axial) plane with slice thickness ranging between 0.5 and 0.625 mm and a maximum temporal resolution of 20 phases per cardiac cycle (83–135 ms). CMR achieves spatial resolution of 1–2 mm in the axial plane with slice thickness of up to 10 mm, however, the temporal resolution is more than double at 50 phases per cardiac cycle (20–50 ms) (42). Moreover, CMR techniques such as phase-contrast MRI enable the assessment of blood stasis by visualising blood velocity inside the LA, in either a 3D region of interest (4D Flow) or in slices (2D Flow), shown in Figure 2. However, 4D Flow suffers from challenges in spatiotemporal resolution and difficulty in measuring PV flows accurately, which currently prevent its full clinical translation (43). While CCT sequences cannot image blood flow, the superior spatial resolution enables detailed visualisation of the LAA anatomy (16). Doses of ionising radiation in CCT image acquisition have considerably decreased in recent years but remain a common limitation of this modality, alongside the administration of contrast agents, particularly in dual-enhanced imaging (33).

### 2.2. Endothelial dysfunction

**FIGURE 3:**
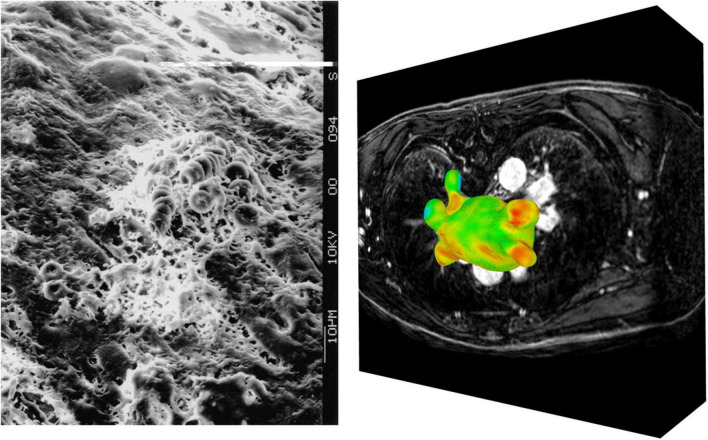
Imaging of endothelial damage. Scanning electron microscopy of a fibrin and erythrocyte rich clot formed in the LAA **(left)** (49). Visualisation of raw LGE intensity map derived from LGE-CMR image stack **(right)**.

The extent of atrial myopathy, such as fibrotic lesions, is correlated with the perpetuation of AF and increased potential for stroke (44). Echocardiography, CCT and CMR can assess AF-related increases in LA volume, a surrogate metric associated with endothelial dysfunction and a higher stroke risk (45). Late gadolinium enhanced MRI (LGE-MRI) can generate detailed maps of LA fibrosis, with increased fibrosis levels associated with increased risk of thrombus formation (46, 47). LGE-MRI maps have also been used to validate LA strain measurements determined from echocardiographic techniques, finding an inverse relationship between strain and myocardial fibrosis, as the formation of stiff fibrotic lesions due to endothelial damage degrades the local contractility of the LA wall (48). The standard for invasive substrate characterisation in patients with AF-related myopathies is electroanatomic mapping, involving transvenous mapping of endocardial electrogram voltages, with regions of low-voltage or electrical silence linked to underlying atrial scar (44). Ex-vivo, scanning electron microscopy has been used to visualise myocardial damage in the LAA, showing that prolonged AF can lead to the creation of endothelial craters, which in turn concurred to form a thrombotic mass, comprised primarily of erythrocytes, at the location of severe endothelial damage (Figure 3) (49).

While these imaging techniques can help to reliably identify LAA thrombi, this is often after their formation. Imaging only provides snapshots of empirical biomarkers of structural changes and thrombogenic states at the time of the scan. Thus, it fails to explain the mechanisms of thrombogenesis or provide prediction of stroke risk, which are key steps in stroke prevention therapy. The most cutting-edge applications of cardiac imaging for AF-related stroke are currently restricted to research environments, limiting their impact. However, the imaging techniques currently available to clinicians have fueled the recent progress in image-based biophysical modelling, showing great promise to identify the mechanistic aspects of thrombogenesis.

## 3. Imaging and modelling of Virchow’s triad

### 3.1. Blood stasis

Computational fluid dynamics (CFD) is an established modelling approach that has been used for decades in (bio)engineering applications (50, 51). By solving the 3D Navier-Stokes equations for fluid motion over a user-defined domain, this technology can quantify blood flow velocity and pressure noninvasively with high spatiotemporal resolution. The accuracy of the model relies on input parameters, such as the fidelity of the anatomical domain segmentation, the conditions defining the behavior of the blood velocity and/or pressure at the domain boundaries (LA wall, PV inlets and mitral valve outlet), and the constitutive parameters of blood (52). Such models can range in complexity from 0D lumped parameter models of blood flow (53, 54) to 3D patient-specific models to replicate realistic cardiac haemodynamics (7, 8, 50, 55–59). Modelling of CFD requires integration in software packages such as CRIMSON, SimVascular and ELMER to perform simulations of cardiovascular flows in 0-3D (60–64).

**FIGURE 4:**
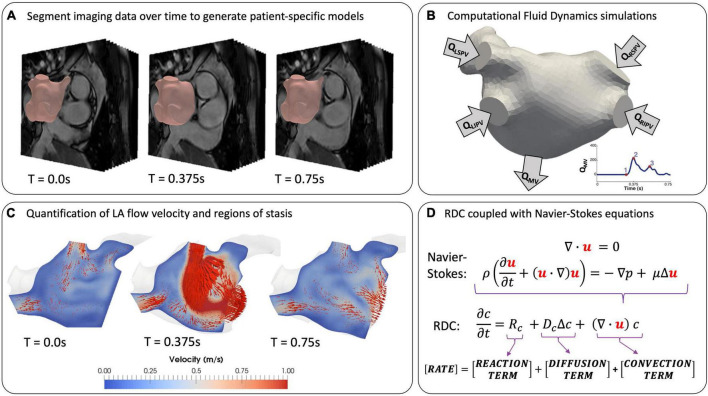
Modelling LA flow from patient data. **(A)** Extracting patient-specific geometries from temporally varying imaging data by segmentation at T = 0 (end-systole). **(B)** Setting boundary conditions on inlets, outlets and LA wall. **(C)** Simulation of LA flow using instantaneous blood flow velocity vectors (red arrows) (57). **(D)** Coupling the equations for fluid motion with reaction-diffusion-convection (RDC) equations to model coagulation in patient-specific geometries.

The most successful applications of CFD to LA flow modelling use 3D patient-specific anatomies and boundary conditions from imaging data, and average values for blood density and viscosity (Figure 4). For example, models can account for the patient-specific myocardial contractility by prescribing the LA wall deformation based on the wall motion tracked from temporally varying imaging data such as Cine-MRI or 4D CCT sequences (Figure 4A). The choice of boundary conditions to personalise the model depends on the availability and quality of the imaging data. 4D CCT series allow for a detailed segmentation of the LA and LAA anatomies at the cost of a low temporal resolution, potentially introducing inaccuracies in wall motion tracking (7, 65), whereas the reverse is true for Cine-MRI sequences (57, 66). Hence, quantitative analyses of shape-dependent LAA haemodynamics are often performed using CCT-based models (56, 67–69). CCT-based CFD studies of the LAA in sinus rhythm (SR) and AF were able to quantify blood stasis by computing the blood residence time inside the LAA, either by releasing a dummy agent concentration in the LAA as a surrogate for SEC (65), or by particle tracking and computation of blood velocity at the LAA entrance (5, 56, 67).

Computational fluid dynamics analyses based on Cine-MRI data yield more accurate measurements of LA cavity flow and motion, but due to poor LAA imaging with this modality, idealised shapes are often used (8, 57, 66). The inflow and outflow conditions are generally treated by prescribing mass flow rates through the PVs and the MV (QPV and QMV in Figure 4B) derived from Doppler echocardiography or 2D Flow data at the valve planes, where available (57, 66, 70). However, the latter is not routinely acquired, and Doppler data is rarely obtained simultaneously with MRI sequences, possibly leading to a mismatch between flow patterns observed in SR or AF using different modalities (6). Several CFD studies achieved successful validation against Doppler echocardiography and 4D Flow data, demonstrating the effectiveness of this approach (7, 43, 65, 70). Currently, all LA flow modelling studies, as summarised in Table 1, have been restricted to small patient cohorts due to the time- and resource-intensive nature of CFD simulations. This limitation, in addition to the lack of a unified validation protocol, has prevented these models from full deployment in the clinical environment.

**TABLE 1:** Models of LA flow.

Study	Study size (N)	Study summary
Vedula et al. (124)	1	4D CCT LA and aortic model
Koizumi et al. (66)	3	CMR-derived LA models with phase contrast MRI for validation
Otani et al. (65)	3	CT-derived LA models with low temporal resolution, validated with TEE
Masci et al. (7)	2	CT-derived LA models with non-patient-specific Doppler data used to prescribe flow through MV
Lantz et al. (70)	3	CT-derived LA and LV models, 4D Flow CMR used for validation
Dillon-Murphy et al. (57)	2	CMR derived LA and LV models, LV volume change used to drive flow through MV
Qureshi et al. (8)	2	CMR derived LA models
Wang et al. (90)	1	Static CCT derived model of LA, coagulation model from Menichini (126) for thrombus growth
Bosi et al. (67)	4	Static CCT-derived models of LA + LAA morphologies
Masci et al. (56)	5	Single CCT-derived LAA, deformed to generate five non-clinically
Fanni et al. (5)	4	CCT-derived models of LAA shapes
Alinezhad et al. (125)	2	CCT-derived models of LA + LAA, AF and SR conditions simulated

P-S, patient-specific; TEE, transoesophageal echocardiography; LV, left ventricle; MV, mitral valve. Upper section denotes studies focusing on LA flow while lower section focuses on LAA haemodynamics.

However, CFD technology is gaining momentum as a clinical tool, with the HeartFlow software package for assessment of coronary artery disease receiving FDA approval, which demonstrates the potential for clinical translation of this approach (71).

### 3.2. Hypercoagulability

AF increases the risk of LA thrombogenesis by inducing a hypercoagulable state due to presence of abnormal thrombogenic protein concentrations (11). Clinical assessment of coagulation function in AF patients relies on the detection of abnormal clotting times, e.g., prothrombin time and international normalised ratio. Coagulability is also evaluated by blood samples to identify biomarkers of elevated coagulation, such as vWf, prothrombin fragment 1+2 and D-dimer (72). Recently, thrombin generation assays have been proposed to reproduce blood coagulation *in vitro*, yet issues with standardisation between centres and lack of clinical validation prompt the search for more robust approaches (73). Blood coagulation is a complex multifactorial process and the metrics derived from these techniques can only provide an indication of underlying abnormalities, without explicitly quantifying the key factors and mechanisms.

In-silico modelling of the coagulation cascade can address some of these limitations by developing a standardised framework to capture the spatiotemporal dynamics of thrombogenic protein concentrations. Such models can also be used to understand the most important parameters responsible for coagulation disorders using sensitivity analyses (74). The complexity of the cascade requires simplifications to strike a balance between practicality and physiological accuracy of the models (75). To achieve this, most studies focus on the final stages of coagulation, where fibrin is generated by the enzymatic cleaving of fibrinogen in blood (76–81). Unlike white thrombi, which form in higher pressure arterial systems and consist mostly of fibrin and platelets, LA thrombi are primarily comprised of fibrin and red blood cells (82). Although platelets are frequently included in extra-cardiac coagulation models, their role in AF-related thrombi and the need to explicitly model them remains unclear. In this case, the effect of red blood cells is accounted for by modelling the spatiotemporal evolution of protein concentrations.

The behavior of clotting factors is commonly modelled by partial differential equations, known as the reaction-diffusion-convection (RDC) equations, which are easy to integrate with CFD models (Figure 4). Two aspects of Virchow’s triad, hypercoagulability and blood stasis, are reflected in the RDC equations. Hypercoagulability is associated with the RD terms, which model the biochemical reactions of thrombin generation and the subsequent stages of coagulation. Blood stasis is represented by the convective term in the RDC equation, with the blood velocity coming directly from the CFD model.

Early studies on coagulability focused on the reactions in the coagulation cascade in the absence of blood flow (79, 83), followed by increasingly complex models with multiple kinetic equations for various proteins in the cascade (77, 78). While reaction equations alone can describe the chemical interactions between proteins at one spatial point, reaction-diffusion models are needed to describe their interplay in space and thrombus growth (83, 84). The more complex reaction-diffusion models include up to 76 equations to capture thrombus formation from initiation to stabilisation, with emphasis on understanding the impact of various proteins on thrombin generation and spatial growth of thrombi (79–81).

**FIGURE 5:**
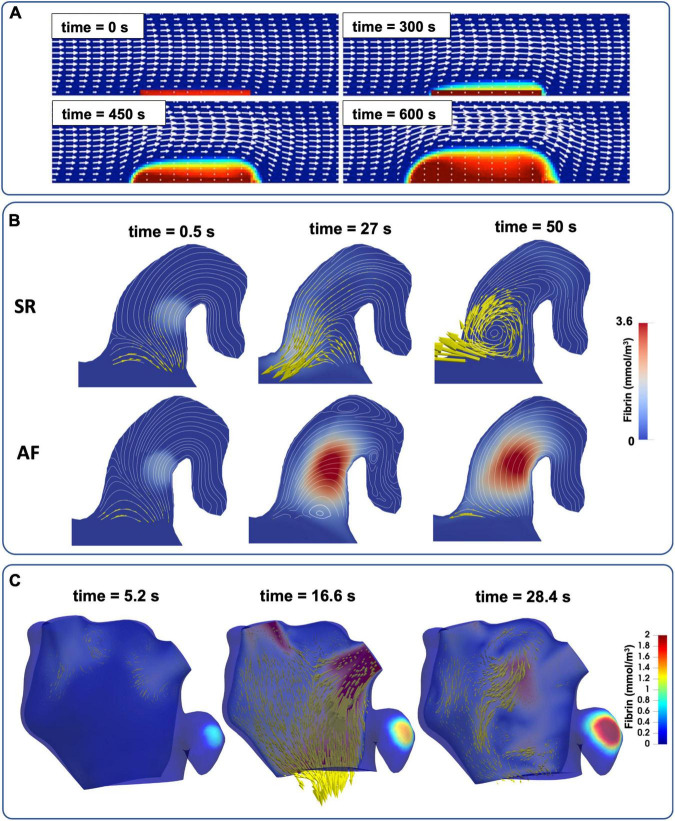
The evolution of full RDC-based thrombus models coupled with blood flow. **(A)** Simple 2D channel with white arrows representing velocity and red regions as bound platelets concentration at the thrombus (77). **(B)** Semi-realistic 2D contracting chicken wing LAA model in both AF and SR conditions with yellow arrows to represent blood velocity and red region as fibrin gel formation (86). **(C)** Patient-specific 3D LA models of LA flow with thrombin concentration representing the thrombus (see also Supplementary video 1).

The complete RDC equations enable the most physiologically accurate representation of in-vivo coagulation by coupling blood flow with the biochemical reactions of the cascade, demonstrating that coagulation under flow conditions indicates an increased threshold for thrombus formation (84). This makes clot solidification less likely to occur without significant thrombin generation due to the surrounding blood flow washing thrombogenic proteins away from site of injury. A pioneering study investigated clot formation using a system of 15 coagulation proteins and platelets linked with CFD simulation of blood flow in a 2D channel setting (77). The polymerisation of fibrin was modelled to create the first two-way coupling between blood flow and thrombus growth, which introduced a resistance to flow local to the clot (Figure 5A). This novel approach has informed subsequent studies in which thrombus growth influences blood flow velocity (78, 85, 86). The study of fibrin and thrombus growth dynamics is a continually growing area of interest in both clinical and engineering research fields (87–89).

More recent studies coupled CFD with the RDC equations, advancing from simple 2D channel flows (Figure 5A) to semi-realistic LAA geometries in AF and SR conditions (Figure 5B) and finally to patient-specific 3D models of the LA in an effort to achieve clinical translation (Figure 5C). Rigid-wall CFD models of the LA based on CCT and Doppler echocardiography data were deployed to assess the thrombogenic role of AF using bound platelets at the area of injury as a biomarker of thrombus formation (90). A similar approach was also used on 3D patient-specific models of the LA in both SR and in AF derived from Cine-MRI data (8, 83, 91) where coagulation dynamics were modelled by initiating a thrombin concentration in two locations (near the PVs and LAA) and the accumulation of thrombin was compared between SR and AF in each case.

After the endothelial injury is repaired in regular hemostasis, the clot is broken down in a process known as fibrinolysis, mediated by tissue plasminogen activator. This essential process dissolves the solid thrombotic mass from the endothelial lining (2). However, this aspect of coagulation, often impaired in AF owing to unregulated thrombus growth, has been represented by RDC equations in general channel flow simulations but remains to be modelled in the fibrillating LA (92).

The modelling of various characteristics of coagulation, summarised in Table 2, has significantly improved knowledge of this complex process. The critical next stage is development of patient-specific coagulation modelling by deriving parameters for the RDC equations. With current technology, these values are measured using thrombin generation assays and mathematical approximations, however significant variability between patients and expensive testing prohibits this from widespread use (93). Furthermore, the precise changes to coagulation mechanisms in AF have not been fully addressed yet (75). Future studies should focus of identifying a small number of key parameters that can be derived from patient measurements, and how these parameters change in AF.

**TABLE 2:** Mathematical models of coagulation.

Study	Key parameters	Study summary
Ataullakhanov et al. (83)	IIa, APC	Threshold nature of coagulation and influence of positive/negative feedback loops on thrombotic protein concentrations
Hockin et al. (79)	34 RDC equations from TF to IIa	Simulated stoichiometric anticoagulants by varying TF concentration to understand its influence on thrombin generation
Panteleev et al. (81)	30+ RDC, platelets	Identified the three stages of thrombin generation (initiation, amplification, and propagation), experimental findings informed rate constants in RDC
Chatterjee et al. (80)	76 RDC species	More than 100 reactions modelled with focus on corn trypsin inhibitor on thrombin generation
Leiderman and Fogelson (77)	IIa, I (Fg), Ia (Fn), Platelets	The first two-way coupling between blood flow and hydraulic resistance due to thrombus porosity. Clot permeability modelled using modified Navier-Stokes equations
Tosenberger et al. (76)	IIa, I (Fg), Ia (Fn), Platelets	Strength of bonds between platelets in core of thrombus creates fibrin cap to prevent further attachment of platelets to slow thrombus growth in flow conditions
Menichini et al. (126)	Generic thrombotic protein and platelets	Altered blood viscosity local to thrombus with application on 3D aortic branch models
Govindarajan et al. (78)	IIa, I (Fg), Ia (Fn), Platelets	Validated numerical simulations with coagulation assays to reproduce physiological measurements

IIa, thrombin; I, fibrinogen; Ia, fibrin; APC, activated protein C; TF, tissue factor.

### 3.3. Endothelial dysfunction

In healthy tissue, the lining of endothelial cells has anticoagulant properties which regulate haemostasis. However, structural remodelling of the LA myocardium in AF exposes sub-endothelial TF, triggering coagulation mechanisms. Identification of LA dysfunction in clinic is primarily based on the presence of increased concentrations of proteins in blood samples, such as vWf and asymmetric dimethylarginine (11).

Computational models of endothelial dysfunction may provide a significant advantage in predicting locations that are prone to thrombus formation. Although this area of research is still in its infancy, existing approaches for personalised identification of cellular remodelling may be translated to thrombogenicity in AF patients (94). The most common approaches for identifying regions of endothelial injury through computational modelling use CFD simulations to assess the time averaged wall shear stress (TAWSS) and oscillatory shear index (OSI), Eqs. 1 and 2. These are indirect metrics for identifying locations where endothelial cellular processes may be altered due to abnormal flow patterns (95, 96). An extension to these metrics proposed a new measurement, known as the endothelial cell activation potential (ECAP), based on the ratio of OSI to TAWSS, with higher ECAP values corresponding to greater risk of endothelial susceptibility, Eq. 3 (97). This was then taken further to calculate a platelet activation potential (PLAP) in Eq. 4 which represents the magnitude of shear rates that a fluid particle accumulates travelling through the fluid domain, and ultimately a metric for thrombus formation potential (TFP) defined as the product of ECAP and PLAP. These measures were then used to assess the thrombogenic risk of different regions in a patient-specific anatomy based on their magnitude and spatial distribution (98). While these methods have primarily been tested on a series of carotid arteries with ECAP with expected orders of magnitude ranging from 0.1 to 10 Pa^–1^ for the ECAP, with further validation, they may also have potential application to the LA in AF. Combining such approaches with the image-based myocardial wall models outlined in Section 3.1 may provide a powerful tool for the prediction of thrombus formation (9, 99, 100).


(1) $$\bar{T\,A\,W\,S\,S}=\frac{1}{T}\,\int_{0}^{T}\left|\tau_{W}\right|\,dt$$

(2) $$O\,S\,I=\frac{1}{2}\,\left(1-\frac{\left|\int_{0}^{T}\tau_{W}\,dt\right|}{\int_{0}^{T}\left|\tau_{W}\right|\,dt}\right)$$

(3) $$E\,C\,A\,P=\frac{O\,S\,I}{\bar{T\,A\,W\,S\,S}}$$

(4) $$P\,L\,A\,P\,\left(x,t\right)=\int_{t-2\,T}^{t}\left|\text{D}\,\left(\text{x}\,\left(\tau \right),\tau \right)\right|\,d\tau$$

Endothelial dysfunction is the most under-explored factor in the modelling of Virchow’s triad. Our incomplete understanding of thrombus formation in AF prompts fundamental questions, such as whether blood stasis or the hypercoagulable state alone would lead to thrombus formation if the endothelium was not compromised, or if the endothelial injury is essential for initiating coagulation in AF. With a lack of consensus on the relative importance of the three factors in Virchow’s triad, further research is required to quantify the role of each factor in thrombogenesis.

## 4. Challenges and future directions

The development of novel medical technologies to improve patient outcomes is the cornerstone of cardiovascular research. Exploration of the latest cardiac modelling techniques may enable a paradigm shift towards low-cost, in-silico technologies to supplement routinely available clinic procedures, as shown by HeartFlow for coronary flow modelling and machine learning algorithms to assess acute stroke severity. Although the cutting-edge modelling approaches described in this Review are still in the early phase of development with limited sample sizes and validation against clinical endpoints, the rapid growth in this field has reached a critical mass in the aim to provide mechanistic tools to improve AF-related stroke risk stratification.

**FIGURE 6:**
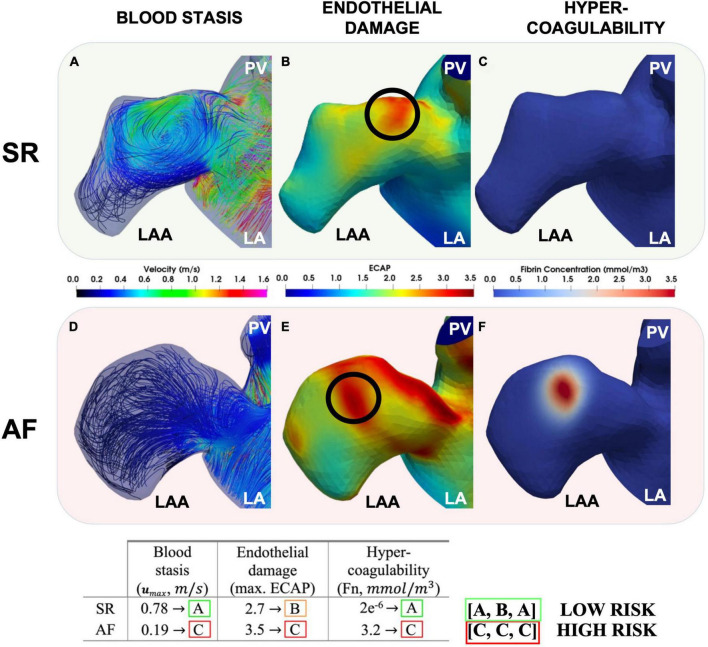
Modelling each aspect of Virchow’s triad. The LAA during SR (top row) and AF (bottom row). Peak LAA blood flow velocities **(A,D)**, ECAP with the location of the maximum ECAP chosen to initiate coagulation [**(B,E)** and black circles] and fibrin thrombus growth due to coagulation after 4 cardiac cycles shown **(C,F)**. Modelling of Virchow’s triad-based mechanistic risk profile also suggested per patient (In Press – Computing in Cardiology 2022).

In future, describing all aspects of Virchow’s triad using image-based modelling may enable a comprehensive evaluation of patient-specific prothrombotic factors (Figure 6). This approach could be used to refine the risk stratification from the CHA_2_DS_2_-VASc score, enabling a true personalisation of anticoagulation drug therapy and optimising of the frequency of imaging follow-up exams based on the prediction of patient outcomes. To achieve this goal, several limitations must be overcome in both fields of imaging and modelling. Cardiac imaging is a leading approach for monitoring risks of thrombus formation in AF patients, but it falls short in evaluating the underlying thrombogenic mechanisms, especially cellular processes in the endothelium, the coagulation cascade, and capturing the intricate structure of the trabeculated LAA. Although new emerging technologies such as 18F-FDG-PET/CT can assess myocardial inflammation by measuring increased fluorodeoxyglucose (FDG) uptake during AF, they remain a niche research area due to limited cost-effectiveness and equipment barriers (101–103).

The significant recent progress in biophysical computer modelling holds potential for enhancing current risk stratification by a comprehensive quantification of patient-specific thrombogenicity based on Virchow’s triad; however, this approach is challenging to perform at the point of care due to significant time-consuming computational expense.

### 4.1. Application of artificial intelligence

**FIGURE 7:**
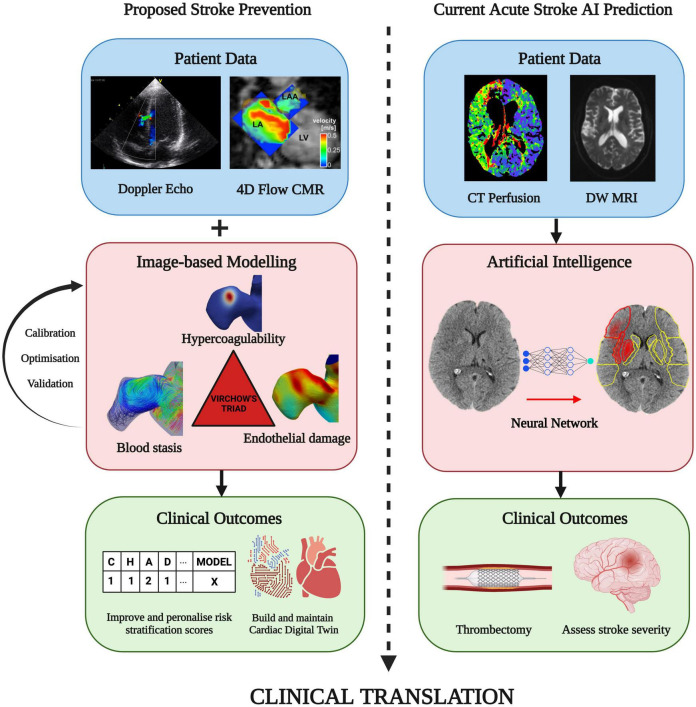
Future direction: A proposed approach on combining patient imaging and modelling of thrombogenic mechanisms in Virchow’s triad. This can improve patient stroke risk stratification and with the aim of preventing stroke prior to thrombus formation. A similar technique is routinely available in clinic using machine learning-based approaches to assess stroke severity and determine the optimal treatment approach, such as performing a thrombectomy (Image courtesy of Brainomix). Integration of machine learning with image-based cardiac modelling can facilitate rapid simulation results and continual updates of a Cardiac Digital Twin for the personalisation and enhancement of AF-related stroke therapy (Figures sourced from 127–129).

Some of these limitations can be overcome by incorporation of machine/deep learning methods to infer prothrombotic biomarkers using image-based biophysical models to accelerate aspects of patient-specific functional assessment (Figure 7) (9, 104–112). An example of this approach is the advent of physics informed neural networks (PINNs) which integrate the PDEs discussed in Section 3.2 as part of the loss function to enable more correct approximations of the solution than earlier forms of machine learning, even with limited data availability (104, 113–115). This development is in line with the Digital Twin vision for precision cardiology which involves creation of a digital representation of the heart updated in real time using data harnessed from electronic health records, imaging data, and wearable technology (116). Combining these approaches by integrating advanced machine learning techniques directly into medical imaging technologies (MRI, CT and Echocardiography devices) to automatically update the *in-silico* Digital Twin may provide the greatest benefit to the patient. With rapid artificial intelligence inference times enabling simulation results in mere seconds directly from patient scans, clinicians may be able to monitor changes in vital patient information to aid in decision making. This approach has grown in availability, with clinically-validated commercial machine learning algorithms (e.g., Brainomix^®^ and RapidAI^®^) frequently being employed in highly time-sensitive evolving stroke cases to optimise triaging and treatment, showing a potential for translation to stroke-prevention for AF patients (Figure 7, right panels) (117–120). Ultimately, combining biophysical modelling accelerated by deep learning approaches in a similar manner may allow for mechanism-based treatment to be tailored to the patient prior to the occurrence of stroke, massively improving patient outcomes and reducing healthcare costs as proposed in Figure 7 (left panels). However, both *in-silico* modelling and machine learning for cardiac thrombogenesis prediction must be developed within a rigorous framework of verification, validation and uncertainty quantification to fulfil regulatory evaluation and become applicable in clinics as a reliable approach to stroke risk assessment (121, 122). A proposed approach for validation of these cutting-edge techniques in a clinical setting may require funding for prospective research studies under controlled conditions with sizeable AF patient cohorts. Parameters from imaging techniques such as Cine MRI, CCT, TEE, Doppler flow, and blood samples would be measured prospectively and used to build computational models. The results of such models can be compared with the patient outcomes and the CHA_2_DS_2_-VASc scores to quantify the accuracy and reliability of *in-silico* technologies for the assessment of patient stroke risk (112).

### 4.2. *In-silico* stroke prevention

Further to the proposed improvements to patient stroke risk stratification, in-silico and imaging techniques can also be used to improve current stroke prevention approaches. Primary treatments for patients at risk of stroke due to AF include prescription of OACs, cardioversion and LAA occlusion therapy. Development of novel OAC’s can be challenging and expensive, however, modelling tools are often employed in the early stages of pharmacological studies using RDC equations (75). Studies have investigated the patient-specific effects of warfarin and heparin on coagulation mechanisms, providing the scope for these tools to be extended to the most recent range of OACs, and development of new drugs for AF patients (93). The choice of whether to cardiovert is based on detection of an LAA thrombus, which can possibly be predicted using the modelling techniques outlined in this Review and ultimately identified by means of routinely available imaging techniques such as TEE. Finally, LAA occlusion (LAAO) is a challenging surgical procedure which involves closing of the LAA entrance to prevent thrombogenesis and is performed when patients are contraindicated to OACs. This technique has warranted pre-surgery virtual implantation using in-silico tools which leverage the patient-specific geometries derived from imaging data. Such interactive modelling can be used to guide and optimise pre-implant planning for clinicians and has shown promising results to reduce risk of device-induced thrombus formation (99, 100).

The field of image-based biophysical modelling is rapidly growing, and novel technologies are continuously being proposed to tackle the greatest challenges facing AF and its associated risk of stroke. The recent developments covered in this Review lay the foundation for the future of this field and eventually, with wider adoption and development, can lead to the translation of such techniques into the clinic for the betterment of the large and growing AF patient population.

## 5. Conclusion

Imaging and modelling of the patient-specific factors and mechanisms of LA thrombus formation can shed light on different aspects of the complex relationship between Virchow’s triad, AF and stroke. Integration of these key aspects will pave the way to develop a new generation of translational models that can enable a cost-effective assessment of patient-specific stroke risks, improving the quality of life and outcomes for the millions of AF patients globally.

## Author contributions

AQ performed literature review and analysis and wrote the manuscript. GL advised on clinical perspectives. DN advised on modelling perspectives. SW advised on imaging perspectives. OA and AV designed, reviewed, and wrote the manuscript. All authors contributed to the article and approved the submitted version.
